# Impact of mild preoperative renal insufficiency on in-hospital and long-term outcomes after off-pump coronary artery bypass grafting: a retrospective propensity score matching analysis

**DOI:** 10.1186/s13019-016-0422-2

**Published:** 2016-02-18

**Authors:** Qiang Ji, LiMin Xia, YunQing Shi, RunHua Ma, ChunSheng Wang, YunQing Mei, WenJun Ding

**Affiliations:** Department of Thoracic Cardiovascular Surgery of Tongji Hospital of Tongji University, Shanghai, 389 Xincun Rd., Shanghai, 200065 P. R. China; Department of Cardiovascular Surgery of Zhongshan Hospital of Fudan University, Shanghai, 180 Fenglin Rd., Shanghai, 200032 P. R. China; Shanghai Institute of Cardiovascular Disease, 180 Fenglin Rd., Shanghai, 200032 P. R. China

**Keywords:** Coronary artery bypass grafting, off-pump, Estimated glomerular filtration rate, Mild renal insufficiency, In-hospital outcomes, Long-term survival

## Abstract

**Background:**

Mild preoperative renal insufficiency is not rare in patients receiving isolated off-pump coronary artery bypass grafting surgery (OPCAB) surgery. However, there is less study aimed to evaluate the impact of mild preoperative renal insufficiency on in-hospital and follow-up outcomes after isolated OPCAB surgery. This single-centre, retrospective propensity score matching study aimed to evaluate the impact of mild preoperative renal insufficiency on in-hospital and long-term outcomes after first isolated OPCAB surgery.

**Methods:**

After propensity score matching, 1236 patients with preoperative estimated glomerular filtration rate (eGFR) of more than 60 ml/min/1.73 m^2^ undergoing first isolated OPCAB surgery from January 2007 to December 2011 were entered into this study and were divided to normal group (eGFR ≥ 90 ml/min/1.73 m^2^, *n* = 618) and mild group (eGFR of 60–89 ml/min/1.73 m^2^, *n* = 618). The in-hospital and long-term outcomes were investigated and retrospectively analyzed.

**Results:**

The 2 propensity score-matched groups had similar baseline and procedural characteristics except the baseline eGFR. Thirty-five patients died during the same hospitalization or within 30 days of operation, with a surgical mortality of 2.8 %. Sixty-seven patients died during follow-up, with a long-term survival of 94.1 %. Univariate factor analysis showed that the 2 propensity score-matched groups have similar rates among in-hospital outcomes. Kaplan-Meier curves displayed a similar in-hospital survival between the 2 groups (χ^2^ = 0.728, *p* = 0.393), while a better long-term survival in patients with normal preoperative renal function compared with mild preoperative renal insufficiency (χ^2^ = 4.722, *p* = 0.030). After Cox proportional model was used, the hazard ratio for long-term mortality in patients with mild preoperative renal insufficiency compared with normal preoperative renal function was 1.72 (95 % CI 1.06–2.83, *p* = 0.032).

**Conclusions:**

Mild preoperative renal insufficiency compared with normal preoperative renal function reduced long-term survival, without evidence of worse in-hospital outcomes.

**Electronic supplementary material:**

The online version of this article (doi:10.1186/s13019-016-0422-2) contains supplementary material, which is available to authorized users.

## Background

Coronary artery bypass grafting surgery (CABG) is recognized as one of the most effective methods for the treatment of coronary heart disease (CHD). Previous studies have demonstrated that preoperative renal failure is an independent risk factor for CABG surgery [1–10]. So, it is crucial to accurate assessment of preoperative renal function. In a majority of previous studies, serum creatinine was usually employed as an indicator for the evaluation of preoperative renal function. However, serum creatinine was gradually recognized to be insufficient to accurately indicate the renal function, because it is affected by some factors such as age, gender, and muscle mass [11, 12]. Furthermore, only when glomerular filtration rate (GFR) decreased by more than 50 % did serum creatinine begin to elevate [11, 12]. And then, its sensitivity was poor in patients with mild to moderate renal insufficiency. Thus, in the clinical practice, preoperative renal function is often overestimated due to serum creatinine as an index of preoperative renal function, especially in the aged patients with mild preoperative renal insufficiency. GFR estimated by equations compared with serum creatinine is more objective and accurate, and is a best indicator of renal function so far [13]. The gold standard for determining the GFR includes inulin clearance rate, isotope measurement and others. However, the detection of the GFR with those methods mentioned above is time-consuming and expensive, and usually requires experience. In recent years, Clinical Practice Guidelines for Chronic Kidney Disease developed by the National Kidney Foundation recommend that some equations (Coekeroft-Gault formula, MDRD formula, etc.) may be used to estimate the GFR [12].

In addition, only patients with preoperative serum creatinine of more than 200 μmol/L were paid attention to in a majority of previous studies. Preoperative serum creatinine of more than 200 μmol/L was considered to be moderate and severe preoperative renal insufficiency. Obviously, moderate and severe preoperative renal insufficiency causes higher incidences of adverse events after CABG surgery [14]. Mild preoperative renal insufficiency is not rare in patients receiving isolated CABG surgery [15]. However, there is less study aimed to evaluate the impact of mild preoperative renal insufficiency on in-hospital and follow-up outcomes after isolated CABG surgery. Whether mild preoperative renal insufficiency had an impact on in-hospital and follow-up outcomes after isolated CABG surgery remained to be determined.

The use of cardiopulmonary bypass and other factors associated with cardiopulmonary bypass have negative impacts on renal function following CABG surgery [16]. By avoiding cardiopulmonary bypass, off-pump CABG (OPCAB) is expected to have less negative impacts on the postoperative renal function [17].

Based on the above analysis, employing eGFR calculated by Cockcroft-Gault formula as an index of preoperative renal function, we reviewed 1236 patients with preoperative estimated GFR (eGFR) of more than 60 ml/min/1.73 m^2^ undergoing first isolated OPCAB surgery, in order to evaluate the impacts of mild preoperative renal insufficiency compared with normal preoperative renal function on in-hospital and long-term outcomes in a single-centre, retrospective propensity score matching study.

## Methods

### Evaluation formula of renal function

The fasting serum creatinine was measured in all included patients within 72 h before surgery and used for estimation of preoperative GFR by using Cockcroft-Gault formula.

Cockcroft-Gault formula as follows:

$$ \mathrm{eGFR} = \left(140\ \hbox{-}\ \mathrm{age}\right) \times \mathrm{weight}/72 \times \mathrm{s}\mathrm{C}\mathrm{r}\ \left(\mathrm{mg}/\mathrm{dl}\right)\ \left[\mathrm{Men}\right] $$

$$ \mathrm{eGFR} = \left(140\ \hbox{-}\ \mathrm{age}\right) \times \mathrm{weight} \times 0.85/72 \times \mathrm{s}\mathrm{C}\mathrm{r}\ \left(\mathrm{mg}/\mathrm{dl}\right)\ \left[\mathrm{Woman}\right] $$

eGFR calculated by Cockcroft-Gault formula was standardized by body surface area.

$$ \mathrm{Body}\ \mathrm{surface}\ \mathrm{area} = 0.007184 \times {\mathrm{weight}}^{0.425} \times {\mathrm{height}}^{0.725} $$

With reference to Clinical Practice Guidelines of National Kidney Foundation, normal renal function was defined as eGFR of 90 ml/min/1.73 m^2^ or more, and mild, moderate and severe renal insufficiency were defined as eGFR of 60 to 89, 30 to 59, and less than 30 ml/min/1.73 m^2^, respectively. This study focused on patients with mild preoperative renal insufficiency (eGFR of 60–89 ml/min/1.73 m^2^) and patients with normal preoperative renal function (eGFR of 90 ml/min/1.73 m^2^ or more).

### Patients

The records of consecutive patients with preoperative eGFR of more than 60 ml/min/1.73 m^2^ undergoing first isolated OPCAB surgery in our center from January 2007 to December 2011 were reviewed. Patients undergoing urgent switch from off-pump to on-pump CABG during surgery were excluded from the study. Any patient with incomplete information from medical records was also excluded. Peri-operative data were respectively obtained from our institutional database and were reviewed using a standard data collection form. Data collection was performed by trained staff (two people). The trained staff, however, did not know the purpose of this study.

From January 2007 to December 2011, a total of 2195 patients received first isolated OPCAB surgery in our centre. Four hundred and sixty-four patients were excluded due to preoperative eGFR of less than 60 ml/min/1.73 m^2^, and 76 patients were excluded due to incomplete information from medical records, leaving 1655 well-documented patients with preoperative eGFR of more than 60 ml/min/1.73 m^2^ (1419 males, with a mean age of 62.5 ± 8.2 years) for data analysis. With reference to Clinical Practice Guidelines of National Kidney Foundation, normal preoperative renal function was found in 731 patients (44.2 %, normal group) and mild preoperative renal insufficiency in 924 patients (55.8 %, mild group). As shown in Additional file 1: Table S1, patients with mild preoperative renal insufficiency compared with normal preoperative renal function had higher proportions of older patients and female, and had lower baseline eGFR, and were more likely to present with hypertension and diabetes mellitus.

Propensity scores were created to quantify the likelihood that a given patient with normal preoperative renal function. Bivariate analyses were conducted to examine differences in baseline characteristics between patients with mild preoperative renal insufficiency (*n* = 924) and patients with normal preoperative renal function (*n* = 731). Propensity scores were then calculated using a multivariate logistic regression model based on the following 12 preoperative characteristics with a significance level of less than 0.20 in bivariate analyses: age, gender, body mass index, smoking, hypertension, diabetes mellitus, hyperlipemia, chronic obstructive pulmonary disease, prior cerebro-vascular accident, recent myocardial infarction, impaired left ventricular function, and emergency procedure. The area under the receiver operating characteristic curve was 0.72 (95 % confidence interval (CI) 0.60–0.79, *p* = 0.02). The Hosmer-Lemeshow goodness for this model was 6.65 (*p* = 0.77). Every patient with normal preoperative renal function was matched with a patient with mild preoperative renal insufficiency with the closest propensity score (within 0.030). Finally, by matching propensity scores, 618 pairs were successfully established in a 1:1 manner (normal group, *n* = 618; mild group, *n* = 618).

### Clinical outcomes

In-hospital outcomes were as follows. Surgical mortality was defined as death occurring during the same hospitalization or within 30 days of the operation. Postoperative myocardial infarction was defined by either the appearance of new Q waves in 2 or more contiguous leads on the electrocardiogram, or an increase in the creatine kinase MB isoenzyme fraction of more than 50U, in concert with an excess of 7 % of the total creatinine kinase level. After OPCAB surgery, any episode of atrial fibrillation that was registered by the monitoring system on a rhythm strip or the 12-lead ECG and lasting for more than 5 min with or without symptoms, was defined as postoperative atrial fibrillation. Intra-aortic balloon pump (IABP) support, postoperative respiratory failure (duration of mechanical ventilation more than 72 h or re-intubation following OPCAB surgery), postoperative pneumonia (a positive result in a sputum culture requiring anti-infective treatment, or chest X-ray diagnosis of pneumonia following cardiac surgery), stroke (new permanent neurological event; early stroke: within 24 h and delayed stroke greater than 24 h postoperatively), redo for bleeding (re-operation to control bleeding within 36 h following initial surgery), red blood cell (RBC) transfusion, acute kidney injury requiring dialysis, and deep sternal wound infection (DSWI) (bone related; any drainage of purulent material from the sternotomy wound and instability of the sternum) were also recorded. The following criteria were employed for the dialysis: anuria, high levels of serum potassium despite diuretic and inotropic support, development of hypervolemia, and acidosis [18].

Postoperative follow-up was completed by clinic visit or telephone. Long-term outcomes included long-term survival and chronic renal failure requiring permanent dialysis.

### Statistical analysis

This study protocol was approved by the ethics committee of Tongji hospital of Tongji University (LL(H)-15-08), and was consistent with the *Declaration of Helsinki*.

Categorical variables are represented as frequency distributions and single percentages. Values of continuous variables are expressed as a mean ± standard deviation (SD). Normally distributed continuous variables were compared using a *Student t-*test, non-normally distributed continuous variables using the *Mann-Whitney U* test, and categorical variables were compared by χ^2^ and *Fisher's exact* test, where appropriate. In-hospital and long-term survival analysis was conducted by Kaplan-Meier method with log-rank test for group comparisons. Estimations of risk were calculated using Cox regression analysis. Potential independent predictors of outcome were identified by univariate Cox regression analyses, and all significant univariate predictors were then entered into the multivariate Cox regression model. All statistical tests were two-sided. Results were considered statistically significant at a level of *p* less than 0.05. All analyses were performed with the SPSS statistical package version 17.0 (SPSS Inc, Chicago, IL, USA).

## Results

### Study population

As shown in Table 1, the 2 propensity score-matched groups had similar baseline characteristics, except the baseline eGFR, which was higher in the propensity-matched normal group (98.0 ± 7.0 ml/min/1.73 m^2^ vs. 75.9 ± 15.4 ml/min/1.73 m^2^, *p* < 0.0001). Patients with mild preoperative renal insufficiency had slightly higher logistic Euro-SCORE as compared to patients with normal preoperative renal function, but no significant difference was found (7.9 ± 2.8 versus 7.9 ± 3.0, *p* > 0.05). Procedural characteristics (including emergent surgery and the number of distal anastomosis) were also balanced between the 2 groups after matching.

**Table 1:** Baseline and procedural characteristics after matching

	Normal group	Mild group	*p* value
	(*n* = 618)	(*n* = 618)	
Age (years old)	62.1 ± 8.1	62.3 ± 8.0	0.1207
Older age (age >65 years)	296 (47.9 %)	302 (48.9 %)	0.7760
Female	80 (12.9 %)	85 (13.8 %)	0.7381
Obesity (BMI >30 kg/m^2^)	194 (31.4 %)	185 (29.9 %)	0.6217
Smoking	308 (49.8 %)	316 (51.1 %)	0.6905
Hypertension	310 (50.1 %)	318 (51.5 %)	0.6904
Diabetes mellitus	198 (32.0 %)	207 (33.5 %)	0.6278
Hyperlipemia	209 (33.8 %)	195 (31.5 %)	0.4305
COPD	73 (11.8 %)	66 (10.7 %)	0.5892
Prior cerebro-vascular accident	56 (9.1 %)	59 (9.5 %)	0.8448
Recent MI	174 (28.2 %)	183 (29.6 %)	0.6156
Impaired left ventricular function	274 (44.3 %)	285 (46.1 %)	0.5677
Extent of CAD			
3 vessel	556 (90.0 %)	560 (90.6 %)	0.7733
2 vessel	62 (10.0 %)	58 (9.4 %)	
LM	190 (30.7 %)	175 (28.3 %)	0.3827
SYNTAX score			
Low: ≤ 22	103 (16.7 %)	106 (17.2 %)	0.4654
Intermediate: 23–32	309 (50.0 %)	294 (47.6 %)	
High: ≥33	206 (33.3 %)	218 (35.2 %)	
Baseline eGFR (ml/min/1.73 m^2^)	98.0 ± 7.0	75.9 ± 15.4	<0.0001
Logistic Euro-SCORE	7.9 ± 2.8	7.9 ± 3.0	0.4136
Emergent	37 (6.0 %)	32 (5.2 %)	0.6205
Number of distal anastomosis	3.4 ± 0.8	3.3 ± 0.8	0.6135

### In-hospital outcomes

As shown in Table 2, no significant difference was found between the 2 propensity score-matched groups in in-hospital outcomes, including stroke, myocardial infarction, atrial fibrillation, IABP support, respiratory failure, pneumonia, redo for bleeding, RBC transfusion, and deep sternal wound infection. Patients with mild preoperative renal insufficiency had slightly higher incidence of acute kidney injury requiring dialysis as compared to patients with normal preoperative renal function, but no significant difference was found (1.3 % vs. 0.3 %, *p* = 0.1080).

**Table 2:** Postoperative outcomes in the matched cohort

	Normal group	Mild group	*p* value
In-hospital			
No. patients	618	618	
Surgical mortality	15 (2.4 %)	20 (3.2 %)	0.4933
Stroke	4 (0.6 %)	7 (1.1 %)	0.5470
Myocardial infarction	19 (3.1 %)	24 (3.9 %)	0.5352
Atrial fibrillation	116 (18.8 %)	123 (19.9 %)	0.6657
IABP support	40 (6.5 %)	44 (7.1 %)	0.7348
AKI requiring dialysis	2 (0.3 %)	8 (1.3 %)	0.1080
Respiratory failure	14 (2.3 %)	22 (3.6 %)	0.2360
Pneumonia	26 (4.2 %)	33 (5.3 %)	0.4237
Redo for bleeding	7 (1.1 %)	9 (1.5 %)	0.8023
RBC transfusion	151 (24.4 %)	168 (27.2 %)	0.2983
DSWI	18 (2.9 %)	25 (4.1 %)	0.3518
Long-term			
No. patients	561	580	
Mortality	24 (4.3 %)	43 (7.4 %)	0.0316
CRF requiring dialysis	0	1 (0.2 %)	1.0000

*IABP* intra-aortic balloon pump, *AKI* acute kidney injury, *RBC* red blood cell, *DSWI* deep sternal wound infection, *CRF* chronic renal failure

Thirty-five patients died during the same hospitalization or within 30 days of operation, with a surgical mortality of 2.8 %. The causes of death are listed in Table 3. The leading causes of death were low cardiac output and infection. Patients with mild preoperative renal insufficiency had slightly higher surgical mortality as compared to patients with normal preoperative renal function, but no significant difference was found (3.2 % vs. 2.4 %, *p* = 0.4933).

**Table 3:** Causes of death in the matched cohort

	Normal group	Mild group
In-hospital		
No. patients	15	20
Low cardiac output	7	9
Infection	5	7
Malignant arrhythmia	3	2
Gastrointestinal bleeding	0	2
Long-term		
No. patients	24	43
Infection	10	17
Heart failure	7	12
Myocardial infarction	2	5
Cancer	2	3
Sudden death	1	2
Pulmonary failure	0	2
Hepatic failure	1	1
Stroke	1	1

### Long-term outcomes

A total of 1141 patients (561 patients in normal group and 580 patients in mild group), accounting for 92.3 %, received follow-up. The mean duration of the observed period in the matched cohort was 54.4 ± 12.3 months in the normal group and 56.5 ± 13.8 months in the mild group. Sixty-seven patients died during follow-up, with a long-term survival of 94.1 %. As shown in Table 3, the main causes of death were infection, heart failure, and myocardial infarction. Patients with mild preoperative renal insufficiency compared with normal preoperative renal function had a higher long-term mortality (7.4 % vs. 4.3 %, *p* = 0.0316). In addition, only one patient in the mild group developed chronic renal failure requiring permanent dialysis.

### Survival and predictors of mortality after OPCAB

**Fig. 1:**
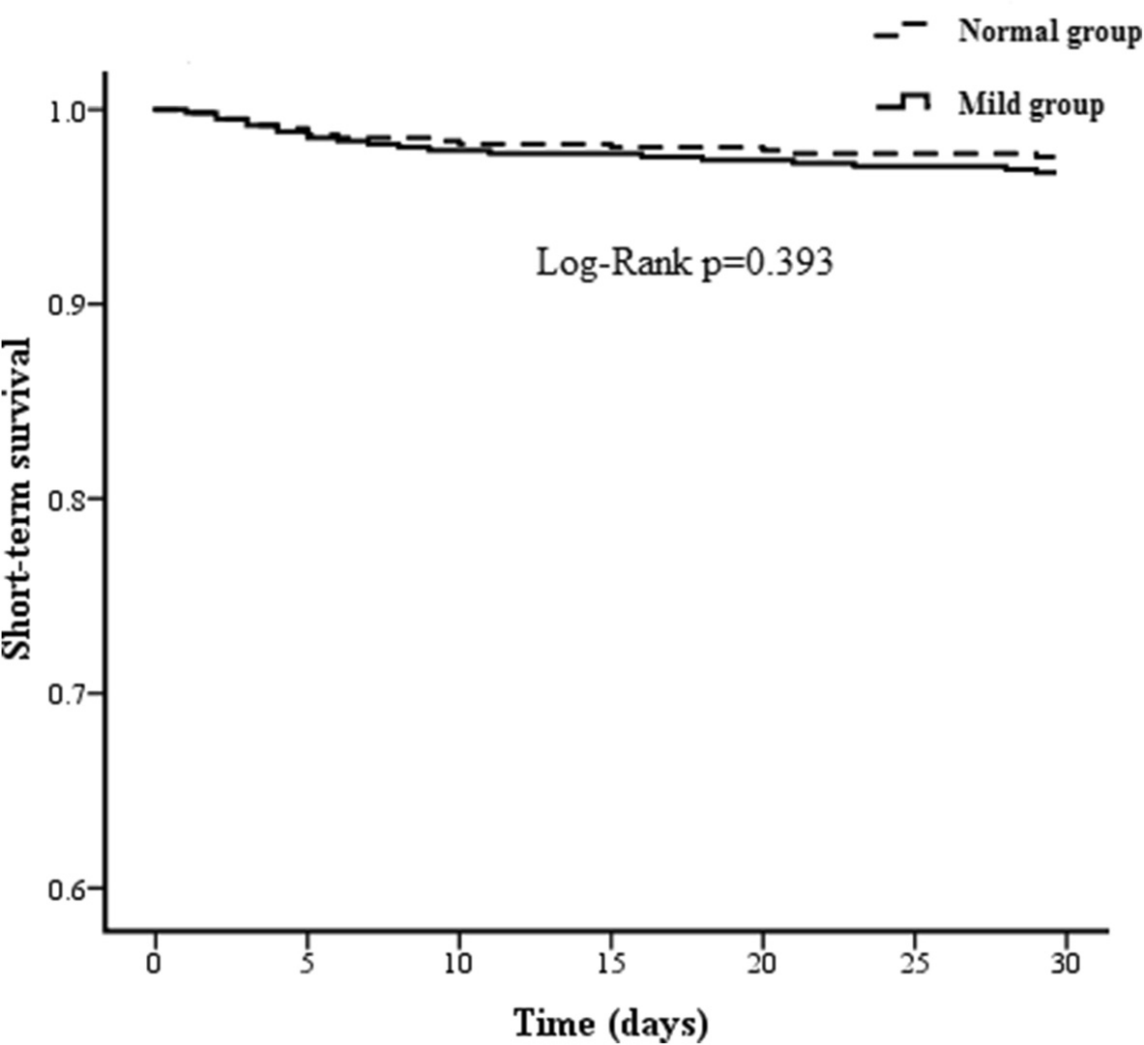
Actuarial curves of in-hospital survival after OPCAB surgery

There was no significant difference in surgical mortality between the 2 propensity score-matched groups (3.2 % vs. 2.4 %, *p* = 0.4933). As shown in Fig. 1, Kaplan-Meier curves displayed a similar in-hospital survival between the 2 groups (χ^2^ = 0.728, *p* = 0.393).

**Fig. 2:**
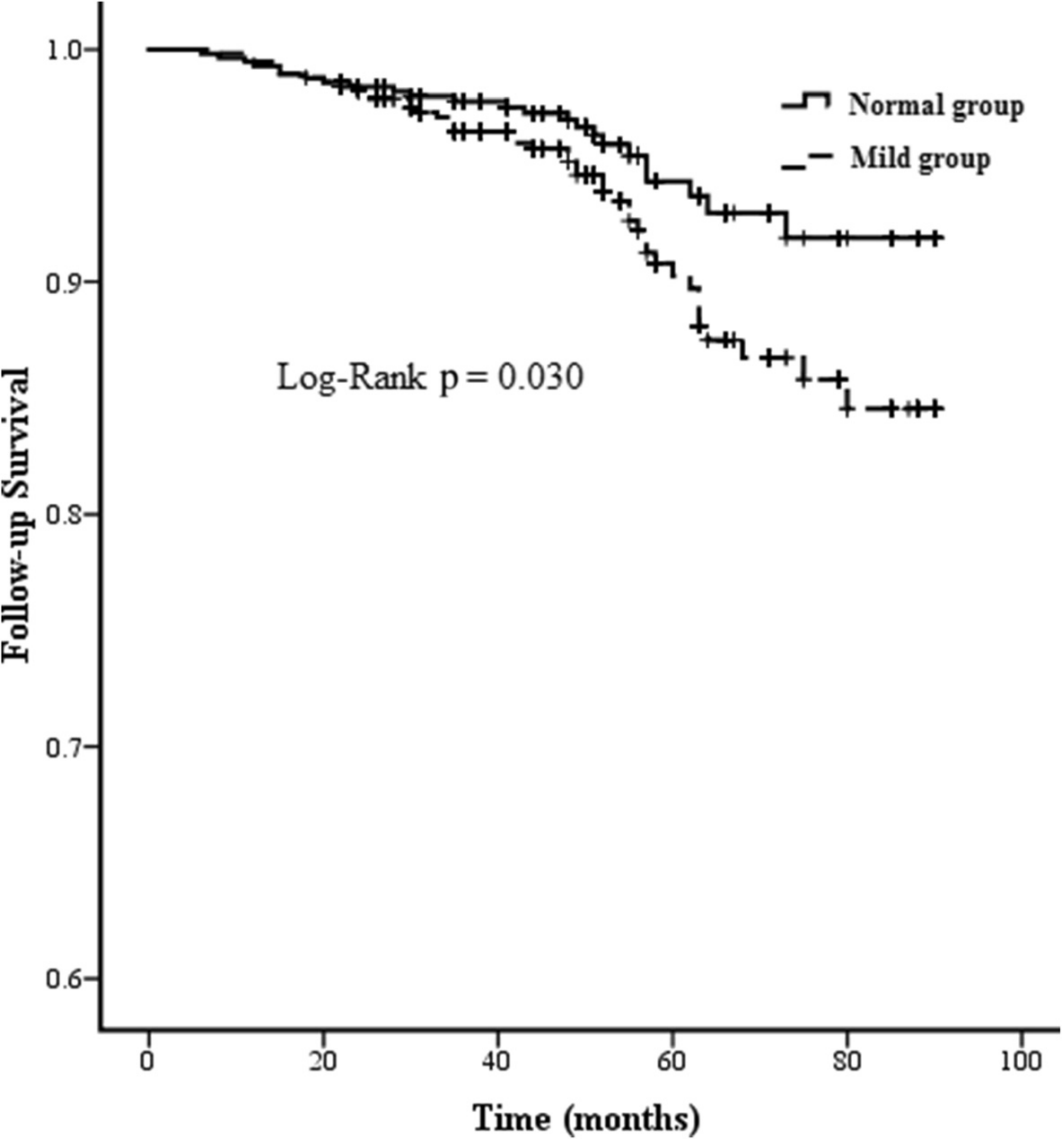
Actuarial curves of long-term survival after OPCAB surgery

During follow-up, 95.7 % patients with normal preoperative renal function and 92.6 % patients with mild preoperative renal insufficiency survived (*p* = 0.0316). As shown in Fig. 2, Kaplan-Meier curves also displayed a better long-term survival in patients with normal preoperative renal function compared with mild preoperative renal insufficiency (χ^2^ = 4.722, *p* = 0.030). Cox regression revealed that grouping (mild preoperative renal insufficiency vs. normal preoperative renal function) was a significant variable related to the long-term survival. After the Cox proportional model was used, the hazard ratio for long-term mortality in patients with mild preoperative renal insufficiency was 1.72 (95%CI 1.06–2.83, *p* = 0.032) (As shown in Table 4).

**Table 4:** Predictors of long-term mortality in the matched cohorts

Variable	HR	95 % CI	*p* value
Grouping (mild group vs. normal group)	1.72	1.06–2.83	0.032
Diabetes mellitus	1.63	1.15–2.52	0.006
Prior cerebro-vascular accident	1.33	1.05–1.92	0.030
Gender (Female vs. male)	1.26	1.08–1.78	0.028
Impaired left ventricular function	1.21	1.05–1.75	0.012
Age (per y)	1.15	1.03–1.60	<0.0001

*HR* hazard ratio, *CI* confidence interval

## Discussion

The important finding of this single-centre, retrospective propensity score matching study was that mild preoperative renal insufficiency compared with normal preoperative renal function reduced the long-term survival after first isolated OPCAB surgery. In this study, after propensity matching, baseline and procedural characteristics were balanced between the 2 groups except the baseline eGFR. Univariate factor analysis showed that patients with mild preoperative renal insufficiency compared with normal preoperative renal function had lower long-term survival (92.6 % vs. 95.7 %, *p* = 0.0316), and Kaplan-Meier curves displayed a better postoperative long-term survival in patients with normal preoperative renal function compared with mild preoperative renal insufficiency (χ^2^ = 4.722, *p* = 0.030). Cox regression revealed that grouping (mild preoperative renal insufficiency vs. normal preoperative renal function) was a significant variable related to the long-term survival. Furthermore, after the Cox proportional model was used, the hazard ratio for long-term mortality in patients with mild preoperative renal insufficiency was 1.72 (95%CI 1.06–2.83, *p* = 0.032), reflecting a 72 % increase in the risk of long-term mortality. This result was consistent with previous studies [10, 15, 19]. The reason why mild preoperative renal insufficiency decreasing long-term mortality after isolated OPCAB surgery deserved to be further studied. Recently, Günday and colleagues [20] conducted a study included 52 consecutive patients with mild preoperative renal dysfunction vs. normal preoperative renal function undergoing uncomplicated CABG surgery, with respect to coronary flow reserve measured by a second harmonic trans-thoracic Doppler echocardiography. They found that although there was a significant increase in the mean coronary flow reserve after CABG surgery compared with baseline coronary flow reserve, patients with mild preoperative renal insufficiency compared with normal preoperative renal function have a significantly lower mean coronary flow reserve after CABG surgery (2.09 ± 0.08 vs. 2.37 ± 0.06, *p* < 0.05). Then they concluded that mild renal insufficiency can produce adverse effects due to deterioration of the micro-vascular bed. So, one reason of mild preoperative renal insufficiency decreasing long-term survival may be that mild preoperative renal insufficiency reduces coronary flow reserve after CABG surgery due to deterioration of the micro-vascular bed.

Another important finding of this single-centre, retrospective propensity score matching study was that patients with mild preoperative renal insufficiency as compared to normal preoperative renal function shared similar rates among in-hospital morbidities and surgical mortality. In this study, univariate factor analysis showed that the 2 propensity score-matched groups have similar rates among in-hospital outcomes, including stroke, myocardial infarction, atrial fibrillation, IABP support, respiratory failure, pneumonia, redo for bleeding, RBC transfusion, deep sternal wound infection, acute kidney injury requiring dialysis, and surgical mortality. And Kaplan-Meier curves also confirmed a similar in-hospital survival between the 2 groups (χ^2^ = 0.728, *p* = 0.393). This result was inconsistent with previous studies [10, 19]. Jyrala and colleagues [10] conducted a study about 885 patients with or without mild preoperative renal dysfunction undergoing on-pump cardiac surgery, with respect to short- and long-term outcomes. They found mild increase in serum creatinine was a marker for patients with increased cardiac risk factors and the risk for poor outcomes. This evidence was in line with our study about postoperative late survival but was different from postoperative short-term outcomes. Reason of this difference can be the study population, regarding that our study only included patients undergoing first isolated OPCAB surgery, and the indicator for the evaluation of renal function, regarding that Jyrala used serum creatinine as the indicator for the evaluation of renal function while we used the eGFR as the indicator for the evaluation of renal function. Howell [19] performed a prospective review of 7621 patients undergoing CABG, valve surgery or combined procedures, with respect to in-hospital mortality and late survival outcome. Employing the eGFR calculated using Cockcroft-Gault formula as the indicator for the evaluation of renal function, they concluded that mild preoperative renal dysfunction is an important independent predictor of in-hospital and late mortality in adult patients undergoing cardiac surgery. This evidence was in line with our study about postoperative late survival but was different from in-hospital mortality. Reason of this difference can be the study population, regarding that our study only included patients undergoing first isolated OPCAB surgery.

There are several limitations of this study. Although using propensity score matching, this study was only a retrospective clinical observational trial in a single center, which may influence the generalizability. A final determination would need a prospective, multi-centre study involving larger sample size. Secondly, the GFR was estimated in this study by using the Cockcroft-Gault formula. The formula provides an acceptable estimate of GFR, but it is not the gold standard for determining GFR. Finally, renal function measurement (eGFR) was based on a single preoperative serum creatinine value, which might fluctuate, particularly in patients with unstable hemodynamics and various medical therapies. This might also affect our findings.

## Conclusions

Mild preoperative renal insufficiency compared with normal preoperative renal function reduced long-term survival, without evidence of worse in-hospital outcomes.

## Additional file

Additional file 1: Table S1. Characteristics of the entire cohort before propensity score matching. (DOC 48 kb)

## Abbreviations

CABG: coronary artery bypass grafting
CHD: coronary heart disease
CI: confidence interval
DSWI: deep sternal wound infection
eGFR: estimated glomerular filtration rate
IABP: intra-aortic balloon pump
OPCAB: off-pump coronary artery bypass grafting
RBC: red blood cell
SD: standard deviation
