# Supplementary material for: Impact of mild preoperative renal insufficiency on in-hospital and long-term outcomes after off-pump coronary artery bypass grafting: a retrospective propensity score matching analysis
Source: J Cardiothorac Surg. 2016 Feb 18;11:30. doi: 10.1186/s13019-016-0422-2 (PMC4757979; doi:10.1186/s13019-016-0422-2)
Supplement: Additional file 1: Table S1. — Characteristics of the entire cohort before propensity score matching. (DOC 48 kb) [file 13019_2016_422_MOESM1_ESM.doc]

**Additional file 1: Table S1. Characteristics of the entire cohort before propensity score matching**

|  | | Normal group  (n=731) | Mild group  (n=924) | *p* value |
| --- | --- | --- | --- | --- |
| Age (years old) | | 61.3± 8.4 | 63.2 ± 8.1 | <0.0001 |
|  | Older age (age >65 years) | 323 (44.2%) | 492 (53.2%) | <0.0001 |
|  | Female | 82 (11.2%) | 154 (16.7%) | 0.0018 |
|  | Obesity (BMI >30 kg/m^2^) | 224 (30.6%) | 271 (29.3%) | 0.5889 |
|  | Smoking | 413 (56.5%) | 549 (59.4%) | 0.2486 |
|  | Hypertension | 333 (45.6%) | 550 (59.5%) | <0.0001 |
|  | Diabetes mellitus | 220 (30.0%) | 361 (39.1%) | 0.0002 |
|  | Hyperlipemia | 241 (32.9%) | 296 (32.0%) | 0.7114 |
|  | COPD | 89 (12.2%) | 103 (11.1%) | 0.5368 |
|  | Prior cerebro-vascular accident | 67 (9.2%) | 90 (9.7%) | 0.7357 |
|  | Recent MI | 206 (28.2%) | 275 (29.8%) | 0.5131 |
|  | Impaired left ventricular function | 325 (44.5%) | 425 (46.0%) | 0.5509 |
|  | Extent of CAD |  |  |  |
|  | 3 vessel | 656 (89.7%) | 834 (90.3%) | 0.7415 |
|  | 2 vessel | 75 (10.3%) | 90 (9.7%) |
|  | LM | 227 (31.1%) | 285 (30.8%) | 0.9573 |
|  | SYNTAX score |  |  |  |
|  | Low: ≤ 22 | 131 (17.9%) | 169 (18.3%) | 0.3564 |
|  | Intermediate: 23-32 | 358 (49.0%) | 428 (46.3%) |
|  | High: ≥33 | 242 (33.1%) | 327 (35.4%) |
|  | Baseline eGFR (ml/min/1.73m^2^) | 98.3±7.2 | 75.9 ±15.6 | <0.0001 |
|  | Logistic Euro-SCORE | 7.8±2.9 | 7.9±2.8 | 0.2179 |
|  | Emergency | 40 (5.5%) | 53 (5.7%) | 0.8308 |
|  | Number of distal anastomosis | 3.4±0.9 | 3.3±0.8 | 0.1197 |

BMI, body mass index; COPD, chronic obstructive pulmonary disease; MI, myocardial infarction; CAD, coronary artery disease; LM, left main trunk disease; eGFR, estimated glomerular filtration rate.
